# Explaining solar forecasts with generative AI: A two-stage framework combining transformers and LLMs

**DOI:** 10.1371/journal.pone.0331516

**Published:** 2025-09-17

**Authors:** Ayesha Siddiqa, Nadim Rana, Wazir Zada Khan, Fathe Jeribi, Ali Tahir

**Affiliations:** 1 Department of Computer Science, University of Wah, Wah, Pakistan; 2 Department of Computer Science, College of Engineering and Computer Science, Jazan University, Jazan, Saudi Arabia; 3 Engineering and Technology Research Center, Jazan University, Jazan, Saudi Arabia; University of Bonab, IRAN, ISLAMIC REPUBLIC OF

## Abstract

Accurate and interpretable solar power forecasting is critical for effectively integrating Photo-Voltaic (PV) systems into modern energy infrastructure. This paper introduces a novel two-stage hybrid framework that couples deep learning-based time series prediction with generative Large Language Models (LLMs) to enhance forecast accuracy and model interpretability. At its core, the proposed **SolarTrans** model leverages a lightweight Transformer-based encoder-decoder architecture tailored for short-term DC power prediction using multivariate inverter and weather data, including irradiance, ambient and module temperatures, and temporal features. Experiments conducted on publicly available datasets from two PV plants over 34 days demonstrate strong predictive performance. The SolarTrans model achieves a Mean Absolute Error (MAE) of 0.0782 and 0.1544, Root Mean Squared Error (RMSE) of 0.1760 and 0.4424, and *R*^2^ scores of 0.9692 and 0.7956 on Plant 1 and Plant 2, respectively. On the combined dataset, the model yields an MAE of 0.1105, RMSE of 0.3189, and *R*^2^ of 0.8967. To address the interpretability challenge, we fine-tuned the Flan-T5 model on structured prompts derived from domain-informed templates and forecast outputs. The resulting explanation module achieves ROUGE-1, ROUGE-2, ROUGE-L, and ROUGE-Lsum scores of 0.7889, 0.7211, 0.7759, and 0.7771, respectively, along with a BLEU score of 0.6558, indicating high-fidelity generation of domain-relevant natural language explanations.

## Introduction

The accelerating global transition toward decarbonized energy systems has positioned solar Photo-Voltaic (PV) technology as a cornerstone of the clean energy revolution. According to the International Energy Agency (IEA), solar PV contributed over 60% of new renewable energy capacity added in 2023, marking it as the fastest-growing electricity source globally [1]. This rapid growth necessitates robust tools for integrating solar power into existing energy infrastructures. One of the critical challenges in this context is the accurate forecasting of solar power generation, which is essential for grid reliability, energy market operations, and intelligent scheduling in smart grid environments.

Unlike conventional power sources, solar energy production is inherently variable and uncertain due to its dependence on meteorological factors such as solar irradiance, ambient temperature, wind speed, and cloud dynamics. These fluctuations introduce significant complexity in prediction tasks, where forecasting models must contend with both temporal nonstationarity and high-dimensional interactions among input features.

Traditional forecasting approaches—ranging from autoregressive statistical models to physics-based simulations—often fall short when capturing these nonlinear dependencies and abrupt changes in solar power time series [2]. Consequently, there has been a paradigm shift toward data-driven approaches, especially deep learning techniques, which have demonstrated superior performance across diverse forecasting horizons. Models such as Convolutional Neural Networks (CNNs), Recurrent Neural Networks (RNNs), and hybrid frameworks have been widely explored for their capacity to learn spatiotemporal patterns directly from raw multivariate data [3,4].

More recently, Transformer-based architectures—originally designed for natural language processing—have shown promise in time series applications due to their self-attention mechanisms, which allow for flexible modeling of long-range dependencies without the sequential bottlenecks associated with RNNs. Notable work by Gao et al. [5] introduced a cross-variable attention-enhanced Transformer that achieved superior results in multivariate PV forecasting tasks. Similarly, Agrawal et al. [6] leveraged the Time Series Transformer for modeling PV cell behavior under varying environmental conditions, while Salman et al. [3] proposed CNN-GRU hybrids with improved short-term accuracy.

However, despite these advances, a persistent challenge remains, i.e., interpretability. Most deep learning models operate as opaque black boxes, offering little insight into the physical or causal mechanisms behind their predictions. As solar forecasting systems are increasingly embedded in critical decision-making processes—such as demand-response planning, real-time dispatching, and market bidding—there is a growing demand for human-interpretable outputs that can support transparent and trustworthy energy management. Explainable AI (XAI) techniques, such as surrogate modeling and attention heatmaps, have been introduced to address this need [4], but often lack domain-specific contextualization or the semantic richness required by stakeholders in real-world applications.

To address the dual challenge of accuracy and interpretability in solar forecasting, this paper introduces **SolarTrans**, a novel two-stage framework that integrates a Transformer-based forecasting model with a generative Large Language Model (LLM) for explainable prediction [7]. By coupling a lightweight encoder–decoder architecture with fine-tuned natural language generation, SolarTrans not only delivers high-fidelity short-term power forecasts but also produces coherent textual explanations that contextualize trends concerning environmental and temporal variables. The framework is trained and evaluated on a high-resolution, real-world dataset comprising 34 days of operational data from two photovoltaic power plants, demonstrating strong generalization and interpretive capabilities. Through extensive experimentation, we show that SolarTrans outperforms baseline models in both predictive accuracy and explanation quality, offering a transparent, deployable solution for intelligent solar energy management. The primary contributions of this paper are summarized as follows:

- We proposed **SolarTrans**, a lightweight Transformer-based encoder–decoder architecture tailored for short-term forecasting of Direct Current (DC) power output
- We developed a two-stage explainable forecasting framework that couples SolarTrans with a generative AI language model, Flan-T5-Solar for the automatic generation of contextualized natural language explanations of predictions
- We fine-tuned the generative AI model Flan-T5 model by constructing a domain-informed explanation dataset using rule-based templates applied to forecast outputs and physical features

The remainder of this paper is organized as follows: the Related Work section reviews prior research; the Proposed Methodology section details the approach used in this study; the Results and Discussion section presents the findings and their interpretation; and the Conclusion section outlines final remarks along with future research directions.

## Related work

Accurate forecasting of PV power generation is critical for the reliable integration of renewable energy into modern power grids. Traditional statistical methods such as ARIMA and exponential smoothing models have been widely used in early-stage forecasting. However, these methods often underperform in real-world PV scenarios due to their limited capacity to handle nonlinearities, temporal dependencies, and complex environmental interactions [8].

To overcome these limitations, recent studies have increasingly adopted deep learning models, which offer enhanced capabilities for learning from high-dimensional, nonlinear, and non-stationary time series data. CNNs and RNNs, particularly Long Short-Term Memory (LSTM) units, have been popular choices due to their strengths in spatial and sequential learning, respectively [9]. Hybrid models that combine both architectures have shown improved performance. Al-Ali et al. [10] proposed a CNN-LSTM-Transformer model that integrates spatial feature extraction with temporal sequence modeling, yielding better short-term irradiance predictions.

Transformer-based models have gained traction for their superior performance in time series domains, owing to their self-attention mechanisms and parallelizable architecture. Gao et al. [5] introduced PV-Client, a Cross-variable Linear Integrated Enhanced Transformer, which incorporates cross-attention to model interactions among meteorological variables, achieving state-of-the-art results in PV power forecasting tasks.

Several studies have highlighted the effectiveness of hybrid and ensemble techniques. Jannah et al. [11] conducted a recent review on solar forecasting models and emphasized the growing shift toward multi-model architectures. These models can capture complementary patterns from different network types, offering enhanced robustness and generalization, particularly across multiple PV sites or weather conditions.

Despite improvements in predictive performance, most deep learning models remain opaque and offer little interpretability, posing challenges for deployment in decision-critical energy management systems. To address this, eXplainable AI (XAI) techniques have been proposed. Rizk et al. [12] presented X-LSTM-EO, a model that couples LSTM forecasting with the Equilibrium Optimizer and utilizes LIME to interpret the influence of input features. Their work demonstrated the feasibility of local surrogate models in making black-box predictions more transparent.

Recent trends also explore model-agnostic interpretability tools such as SHAP, attention-based saliency maps, and surrogate rule-based explanations. However, these methods often fail to deliver explanations that are easily interpretable by non-technical stakeholders or lack linguistic expressiveness.

The integration of LLMs into time series applications is an emerging area, particularly for generating contextualized, human-readable explanations. While still nascent in energy domains, early works from related fields (e.g., finance and healthcare) have shown promise in using models like Text-to-Text Transfer Transformer (T5) and Generative Pre-trained Transformer (GPT)—both of which are built upon the Transformer architecture introduced by Vaswani et al. [13]—to translate numerical forecasts into narrative descriptions. These developments open avenues for combining deep forecasting architectures with LLMs to support transparency, trust, and stakeholder communication—an approach explored in this work.

Significant progress has been made in advancing forecasting accuracy using deep and hybrid models, particularly Transformers. However, the integration of domain-aware explainability remains an underexplored area. Existing methods either focus solely on accuracy or apply generic XAI tools that lack contextual depth. This paper addresses this gap by proposing a unified framework that combines Transformer-based forecasting with generative language modeling to produce accurate and interpretable solar power predictions.

## Proposed methodology

**Fig 1:**
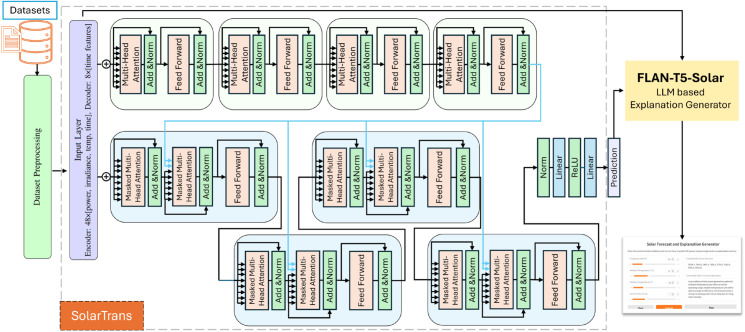
The architecture of the proposed SolarTrans for solar power prediction LLM-based generated explanations.

This section details the proposed hybrid architecture for short-term solar power forecasting with integrated natural language explanations. The framework consists of two primary modules: (i) a Transformer-based deep learning model (SolarTrans) for multi-horizon direct current (DC) power prediction, and (ii) a fine-tuned large language model (LLM) for post-hoc interpretability. The end-to-end system workflow is illustrated in Fig 1.

### Problem formulation

Let ${𝐗}_{t}\in {\mathbb{R}}^{n\times d}$ denote the input multivariate time series, where *n* is the encoder window size and *d* represents the number of input features. The objective is to forecast the DC power output ${𝐲}_{t+1:t+\tau }\in {\mathbb{R}}^{\tau }$ over *τ* future time steps. The model learns a mapping as represented in Eq 1.

$${\hat{𝐲}}_{t+1:t+\tau }=f_{\theta }({𝐗}_{t-n+1:t},{𝐓}_{t+1:t+\tau })\;\text{with}\;{ℒ}_{\text{forecast}}=\frac{1}{\tau }\sum_{i=1}^{\tau }|\;{\hat{y}}_{t+i}-y_{t+i}|$$ (1)

where ${\hat{𝐲}}_{t+1:t+\tau }$ is the predicted DC power sequence, **T** comprises known temporal covariates (e.g., hour of day, day of week, month), and $f_{\theta }$ denotes the forecasting function parameterized by *θ*.

### Data preprocessing and normalization

To capture both periodic and environmental influences, we incorporate calendar-based features (e.g., hour, day of week, month) alongside meteorological and operational variables (e.g., solar irradiance, ambient temperature, module temperature, and prior DC power). Given the site heterogeneity across inverters, we normalized each feature using z-score standardization on a per-inverter basis using Eq 2:

$$z=\frac{x-\mu_{\text{inv}}}{\sigma_{\text{inv}}},$$ (2)

where $\mu_{\text{inv}}$ and $\sigma_{\text{inv}}$ denote the inverter-specific mean and standard deviation. This ensures scale invariance and improves model generalization across devices.

Input sequences are constructed using a sliding window mechanism, with encoder and decoder lengths set to 48 and 8 time steps, respectively. Each training instance consists of a historical sensor window, associated future time covariates, the ground truth DC power trajectory, and the inverter identifier.

### SolarTrans: Transformer-based forecasting model

The proposed SolarTrans model leverages a sequence-to-sequence Transformer architecture specifically designed for multi-horizon forecasting of DC power in solar PV systems. It jointly incorporates historical multivariate sensor measurements and future time-based features to produce robust point forecasts. The model receives two distinct input streams: (i) past sensor observations ${𝐗}_{t-n+1:t}\in {\mathbb{R}}^{n\times d_{x}}$ processed by the encoder, and (ii) future time metadata ${𝐓}_{t+1:t+\tau }\in {\mathbb{R}}^{\tau \times d_{t}}$ processed by the decoder. Both inputs are linearly projected into a shared embedding space of dimension $d_{\text{model}}=64$ and augmented with learnable positional encodings to retain temporal order.

To enforce causal structure in autoregressive decoding, a *causal mask* is applied to the decoder’s self-attention layers, ensuring that predictions at time step t+k cannot access future positions t+k+1,…,t+τ. The Transformer core comprises *L* = 4 stacked encoder and decoder layers, each employing *H* = 8 attention heads and dropout regularization (*p* = 0.1). The final decoder representation is passed through a feedforward prediction head with layer normalization and residual connections to produce the output sequence ${\hat{𝐲}}_{t+1:t+\tau }$. SolarTrans captures both short-term temporal dependencies and long-range contextual patterns, making it well-suited for high-resolution solar power forecasting. Table 1 outlines the layer-wise tensor shapes and data flow in the proposed SolarTrans model. Meanwhile, Table 2 details the internal architecture of the Transformer encoder and decoder layers employed for solar power forecasting.

**Table 1:** Layer-wise architecture detail of the proposed SolarTrans model.

Stage	Layer	Output Shape	Input From
**Encoder Input Pipeline**			
E0	Input Sequence	$\left(B,\;T_{\text{enc}},\;8\right)$	input features (weather + time)
E1	Linear Projection	$\left(B,\;T_{\text{enc}},\;64\right)$	E0
E2	+ Positional Encoding	$\left(B,\;T_{\text{enc}},\;64\right)$	E1
**Decoder Input Pipeline**			
D0	Input Sequence	$\left(B,\;T_{\text{dec}},\;4\right)$	Time features
D1	Linear Projection	$\left(B,\;T_{\text{dec}},\;64\right)$	D0
D2	+ Positional Encoding	$\left(B,\;T_{\text{dec}},\;64\right)$	D1
**Transformer Core**			
T1	Transformer Encoder (×4)	$\left(B,\;T_{\text{enc}},\;64\right)$	E2
T2	Transformer Decoder (×4)	$\left(B,\;T_{\text{dec}},\;64\right)$	D2 + T1
**Prediction Head**			
H1	LayerNorm	$\left(B,\;T_{\text{dec}},\;64\right)$	T2
H2	Linear + ReLU + Dropout	$\left(B,\;T_{\text{dec}},\;64\right)$	H1
H3	Linear Output	$\left(B,\;T_{\text{dec}},\;1\right)$	H2
**Output**	Predicted Sequence	$\left(B,\;T_{\text{dec}}\right)$	H3

**Table 2:** Transformer encoder-decoder block details in the SolarTrans.

Block	Component	Details
T1	Multi-Head Self-Attention	MultiheadAttention(64, 8), Dropout=0.1
	Feed-Forward Network	Linear(64→2048) → ReLU → Linear(2048→64), Dropout=0.1
	Layer Normalization	2 × LayerNorm(64) (post-attn and post-ffn)
	Residual Connections	Applied after attention and FFN
T2	Masked Multi-Head Attention	Masked MultiheadAttention(64, 8), Dropout=0.1
	Encoder-Decoder Attention	MultiheadAttention(64, 8), Dropout=0.1
	Feed-Forward Network	Linear(64→2048) → ReLU → Linear(2048→64), Dropout=0.1
	Layer Normalization	3 × LayerNorm(64) (post each sublayer)
	Residual Connections	After all sublayers

### Flan-T5-Solar: Domain-adaptive explanation module

In addition to generating accurate numerical DC power forecasts, the SolarTrans framework incorporates a domain-adapted language model, Flan-T5-Solar, to produce human-interpretable explanations. This module enhances the transparency of the forecasting process by contextualizing the predicted power output concerning recent weather conditions and operational trends. Flan-T5-Solar is based on Flan-T5-Base. Flan-T5 [14] is a 250M-parameter encoder-decoder language model derived from Google’s T5 architecture [15], optimized through large-scale instruction finetuning across 1,800+ diverse tasks. This finetuning enables strong zero-shot and few-shot generalization, allowing the model to outperform its original T5 counterpart. This language model is fine-tuned on structured prompts that encode the relevant environmental conditions and numerical forecast outputs.

Input Construction: Each explanation input prompt is constructed using denormalized features derived from the SolarTransformer output. Specifically, the input text includes the latest values of irradiance, ambient temperature, module temperature, the forecast time window, and the predicted DC power sequence. These features are concatenated into a structured, natural-language sentence to ensure that the model receives consistent domain cues. For example:

Irradiance: 910 W/m^2^, Ambient Temp: 27.3^°^C, Module Temp: 35.0^°^C, Forecast Hours: [6, 7, 8, 9, 10, 11], Predicted DC Power: [4.3, 4.8, 5.1, 5.4, 5.3, 5.0] kW.

Rule-Based Explanation Generation: To produce physically plausible explanations, we first employ a rule-based template (Algorithm 1) that conditionally appends statements based on irradiance levels, temperature ranges, and power output trends. This ensures consistency with solar physics principles, such as preventing high generation predictions at night or during zero irradiance.

**Algorithm 1. Rule-based solar forecast explanation generation.**



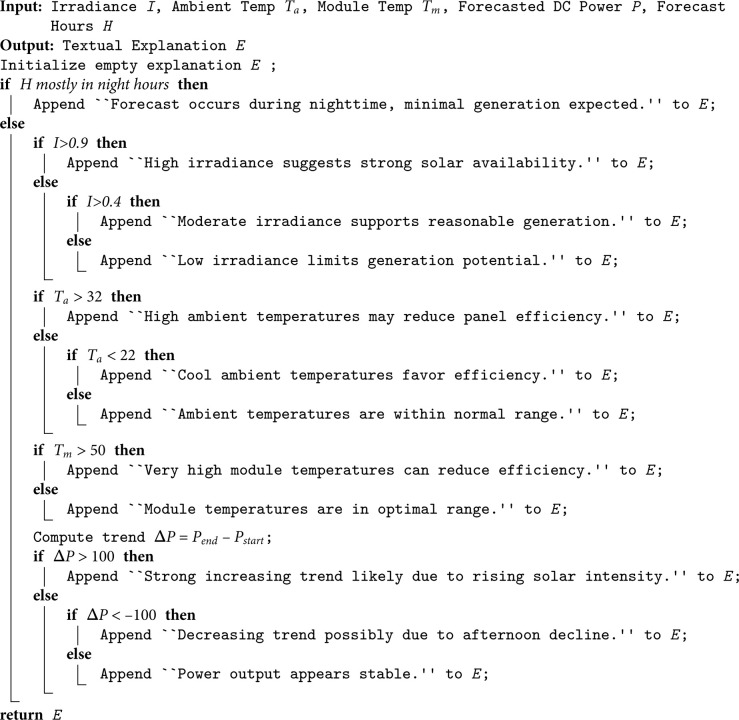



The Flan-T5-Base model is then fine-tuned using a large collection of input-output pairs generated with the rule-based module. We prepare training data in JSONL format, where each record contains the structured input prompt and its corresponding expert-style explanation. This domain-specific fine-tuning enables Flan-T5-Solar to generalize beyond the training examples and generate robust, context-aware explanations for unseen data. During inference, the SolarTransformer’s denormalized outputs are passed to Flan-T5-Solar as input prompts. The model generates concise text rationales that reflect how the forecast aligns with weather dynamics, such as high irradiance driving increased output or temperature conditions affecting panel efficiency. This hybrid approach improves stakeholder trust by making model predictions interpretable in operational settings. Fig 1 illustrates the complete system flow from numerical forecasting to explanation generation. This integrated approach improves transparency and stakeholder trust by providing both quantitative predictions and actionable, context-aware justifications.

### Inference pipeline

At inference time, the system operates in two sequential stages: (i) the SolarTrans module predicts DC power output over the next τ=8 time steps using recent observations and time features, and (ii) the LLM module generates a corresponding textual explanation. This hybrid pipeline yields both accurate numerical forecasts and interpretable rationales, supporting actionable insights for solar farm operators and grid stakeholders. The complete SolarTrans framework combines a custom Transformer encoder-decoder for multivariate time-series forecasting with a domain-adapted Flan-T5 language model to generate interpretable textual explanations.

## Results and discussion

### Dataset description and experimental setup

This study utilizes the publicly available Solar Power Generation Data dataset from Kaggle [16], which contains generation and weather sensor data collected from two solar power plants located in India over 34 days with observations recorded at 15-minute intervals. The overview of the dataset is given in Table 3. Each plant’s dataset is divided into two components, i.e., Generation Data and Weather Sensor Data. The Generation Data includes power output metrics from individual inverters, such as DC power output, AC power output, Daily Yield, Total Yield. Each plant comprises 22 unique inverters, identified via the SOURCE_KEY field, resulting in granular inverter-level monitoring. The Weather Sensor Data provides synchronized environmental variables measured at the plant level, including: Irradiation, Ambient Temperature and Module Temperature. To integrate generation with environmental context, datasets were merged on DATE_TIME and PLANT_ID. Timestamp parsing ensured consistent temporal alignment, and malformed entries were discarded.

**Table 3:** Summary of solar power generation dataset.

Attribute	Description
Data Source	Kaggle Public Dataset [16]
Plants	2 (Plant 1 and Plant 2)
Inverters	44 total (22 per plant)
Time Span	15 May 2020 – 17 June 2020
Temporal Resolution	15-minute intervals
Total Records	136,472 samples
Plant 1 Records	68,774 samples
Plant 2 Records	67,698 samples
Features	Commom: [Date Time, Plant ID, SOURCE KEY], Generation Data: [DC power, AC Power, Daily Yield, Total Yield], Weather Sensor data: [Irradiation, Ambient Temperature, Module Temperature]

#### Dataset preprocessing.

The raw solar generation and weather datasets were first parsed with plant-specific datetime formats and merged using the common fields DATE_TIME and PLANT_ID. The final combined dataset consists of 136,472 records (68,774 from Plant 1 and 67,698 from Plant 2), covering two plants and a total of 44 inverters (22 per plant). The preprocessing pipeline included the following steps. The missing values in continuous features (dc power, irradiation, ambient_temp, and module_temp) were imputed using forward-fill within each inverter’s time series. Additional temporal features such as hour, day, weekday, and month, were engineered from the datetime index to capture diurnal and seasonal patterns. The continuous variables were standardized using the StandardScaler independently for each inverter to preserve device-specific dynamics and to avoid information leakage across groups.

The preprocessed time series was segmented into sliding input-output sequences tailored for sequence-to-sequence forecasting. Each encoder input spans 48 time steps (equivalent to 12 hours at 15-minute resolution) and includes both continuous meteorological variables and timestamp-based features. The decoder input spans the following 8 time steps (i.e., 2 hours) and includes only the known time-based features. The target sequence comprises actual DC power output values over the decoder horizon. The full sequence dataset was split into training (70%), validation (10%), and test (20%) subsets using random_split with a fixed seed for reproducibility. Each sample retains its inverter ID to support future grouping or domain adaptation strategies.

The proposed SolarTrans model was implemented using PyTorch on the Kaggle platform. The model utilizes a dual-input architecture with separate linear projections for encoder and decoder inputs, followed by a Transformer encoder-decoder core. The key hyperparameters are listed in Table 4. The selection of input features was driven by a combination of domain knowledge, relevance to solar power forecasting, and data quality considerations. The proposed SolarTrans model uses a focused set of eight features: DC Power (target variable), Irradiation (proxy for solar insolation), Ambient temperature (affects inverter and panel efficiency), Module temperature (directly impacts photovoltaic cell performance), hour, day, weekday, month (to capture diurnal and seasonal patterns) Features such as AC Power, Total Yield, and Daily Yield are highly correlated with DC Power and are derived variables. Some metadata fields (e.g., Source Key, Plant ID) are used for grouping or domain-specific modeling but are not meaningful as numerical inputs to a forecasting model. Time-based features (hour, day, weekday, month) are known and embedded in a temporal model, helping capture seasonality without depending on future weather predictions.

**Table 4:** Hyperparameters of the proposed model.

Hyperparameter	Value
Embedding dimension	16, 32, **64**
Number of attention heads	2, 4, **8**
Transformer layers (encoder and decoder)	1, 2, **4**
Forecast horizon	8 time steps (2 hours)
Batch size	64
Optimizer	AdamW
Learning rate	5e−4
Weight decay	1e−3
Epochs	60
Loss Function	MSE

Model training was performed using early stopping based on validation loss. The SolarTrans model was trained on the Kaggle platform using a dual NVIDIA T4 GPU setup (T4×2), which provides 32 GB of total GPU memory. The experiments were carried out within the Kaggle-hosted Jupyter Notebook environment, using PyTorch for the implementation of the model and the acceleration of training.

### Quantitative evaluation

We evaluated the model using three commonly adopted regression metrics: Mean Absolute Error (MAE) measures the average magnitude of errors. Root Mean Squared Error (RMSE) penalizes larger errors more significantly. The coefficient of Determination (*R*^2^) measures how well predictions explain the variance in ground truth.

**Fig 2:**
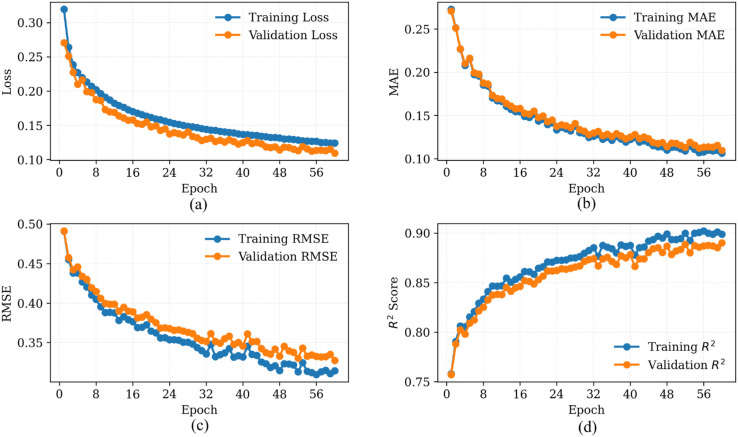
Training and validation loss curves over 60 epochs on combined datasets.

**Fig 3:**
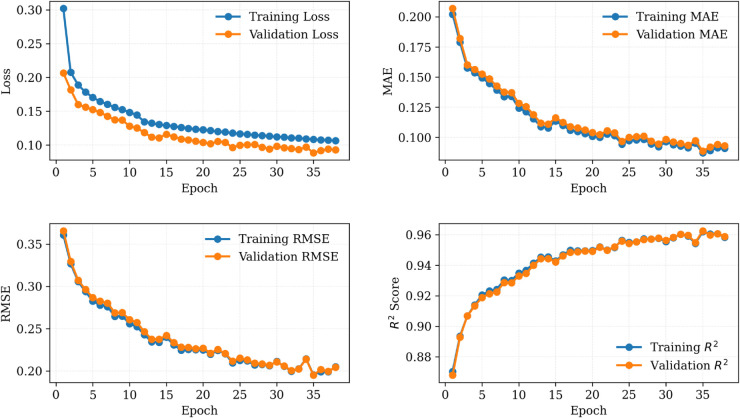
Training and validation loss curves using Plant 1 dataset.

**Fig 4:**
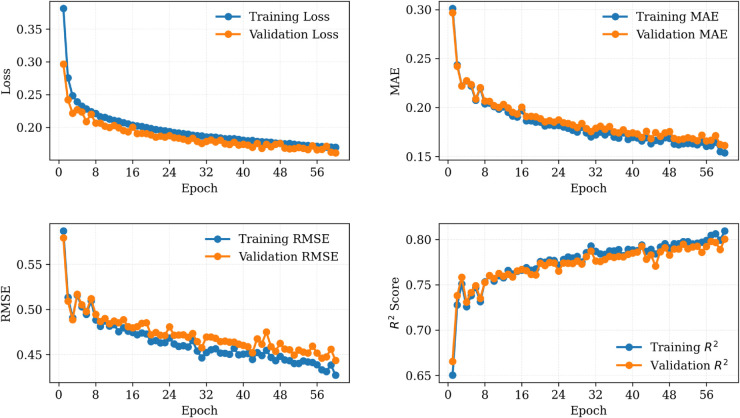
Training and validation loss curves using Plant 2 dataset.

Fig 2 shows plots of the training and validation losses, MAE, RMSE, and *R*^2^ across epochs on the combined dataset from Plant 1 and Plant 2. The model converged smoothly, with validation loss plateauing after approximately 60 epochs. Similarly, Fig 3 and Fig 4 demonstrate the performance of the proposed SolarTrans model using datasets from Plant 1 and Plant 2, respectively.

**Fig 5:**
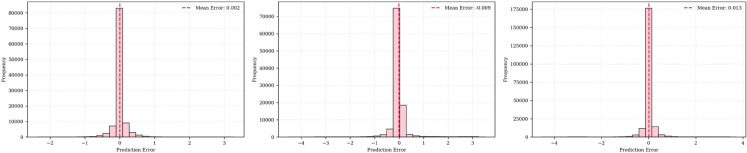
Error distribution of SolarTrans model across Plant 1, Plant 2, and Combined datasets.

The error distribution analysis of the SolarTrans model across different datasets is shown in Fig 5, and it reveals insightful patterns about the model’s generalization ability and site-specific performance. On Plant 1, the model achieved a notably low error concentration around 0.002, indicating highly precise forecasts with minimal deviations. In contrast, SolarTrans showed a wider error spread with a peak around 0.009 on the Plant 2 dataset. While still within an acceptable range, this increased error suggests the presence of more noise, variability, or less predictable weather conditions at that location. Additionally, potential discrepancies in sensor calibration or maintenance quality might contribute to this elevated uncertainty. The higher mean error for Plant 2, as seen in both MAE and RMSE values, is consistent with the broader error distribution. The combined dataset yielded an error peak at 0.013, slightly higher than either individual plant. This is expected due to increased heterogeneity in the merged data, where site-specific conditions, such as geographic and climatic differences, introduce more complex variability. Despite this, the SolarTrans model maintained robust performance. Table 5 reports the performance of the proposed model test sets of Plant 1, Plant 2, and Combined datasets.

**Table 5:** Performance of SolarTrans.

Dataset	MAE	RMSE	R^2^
Plant 1	0.0782	0.1760	0.9692
Plant 2	0.1544	0.4424	0.7956
Combined	0.1105	0.3189	0.8967

**Fig 6:**
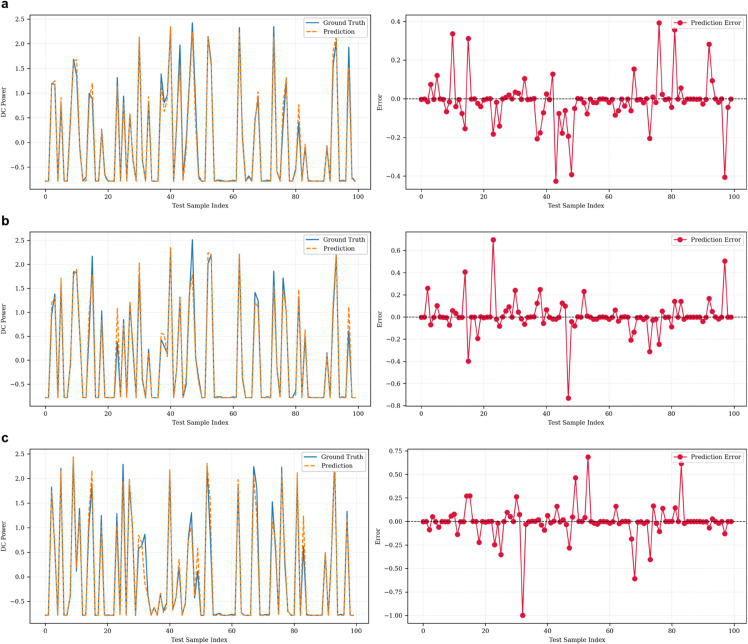
Prediction and error analysis of SolarTrans on the Plant 1 dataset at forecasting steps (a) 1, (b) 4, and (c) 8.

**Fig 7:**
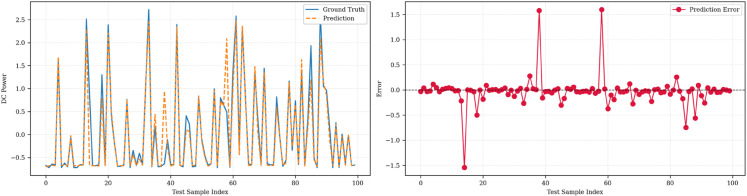
Prediction and error analysis of SolarTrans on the Plant 2 dataset at forecasting step 1.

**Fig 8:**
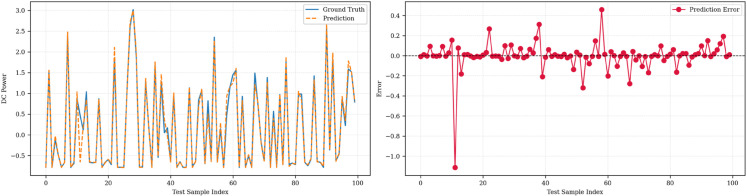
Prediction and error analysis of SolarTrans on the Combined dataset at forecasting step 1.

Fig 6, Fig 7, and Fig 8 illustrate the prediction performance of the SolarTrans model for the first forecast horizon across Plant 1, Plant 2, and the combined dataset, respectively. Prediction and error analysis of SolarTrans on the Plant 1 dataset is illustrated at forecasting steps 1,4,8 in Fig 6. The left subplot shows the model predictions against ground truth for the first 100 test samples, while the right subplot presents the corresponding signed prediction error. The model demonstrates high accuracy and low error variance across short- and long-term horizons, consistent with the low MAE and RMSE observed in this dataset. The prediction error plots highlight the robustness of SolarTrans on Plant 1 and the challenges posed by data heterogeneity in the combined and Plant 2 datasets. The predicted values closely follow the ground truth in Plant 1, with minimal error fluctuations, aligning with its low MAE. Plant 2, however, exhibits greater deviation and residual error variance, particularly around abrupt transitions in DC power output, likely due to more volatile weather conditions or noisier sensor data. The combined dataset demonstrates intermediate behavior, with slightly smoothed errors but wider variance compared to Plant 1.

**Fig 9:**
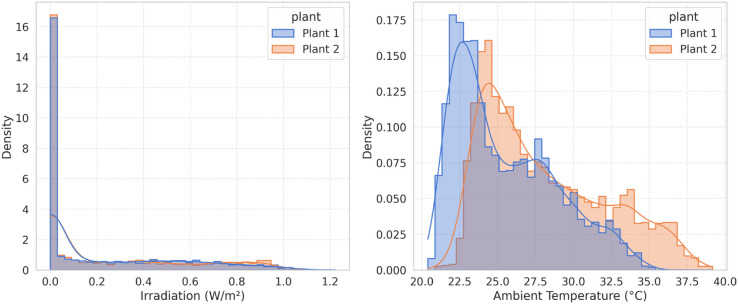
Irradiation and Ambient Temperature Distribution at Plant 1 and Plant 2.

To investigate the impact of environmental variability on model performance, the distributions of irradiation and ambient temperature for both plants are compared as shown in Fig 9. The histograms with overlaid probability density estimates reveal that Plant 2 exhibits broader and flatter distributions, particularly in ambient temperature. This indicates that Plant 2 operates under more diverse and volatile environmental conditions. In contrast, Plant 1 shows more concentrated distributions, suggesting a more stable operating environment. These patterns are consistent with the observed higher residual variance in DC power predictions for Plant 2, especially during periods of abrupt change. Thus, these plots provide visual confirmation that environmental volatility, rather than data noise, is a plausible driver of the residual error patterns seen in Plant 2.

**Fig 10:**
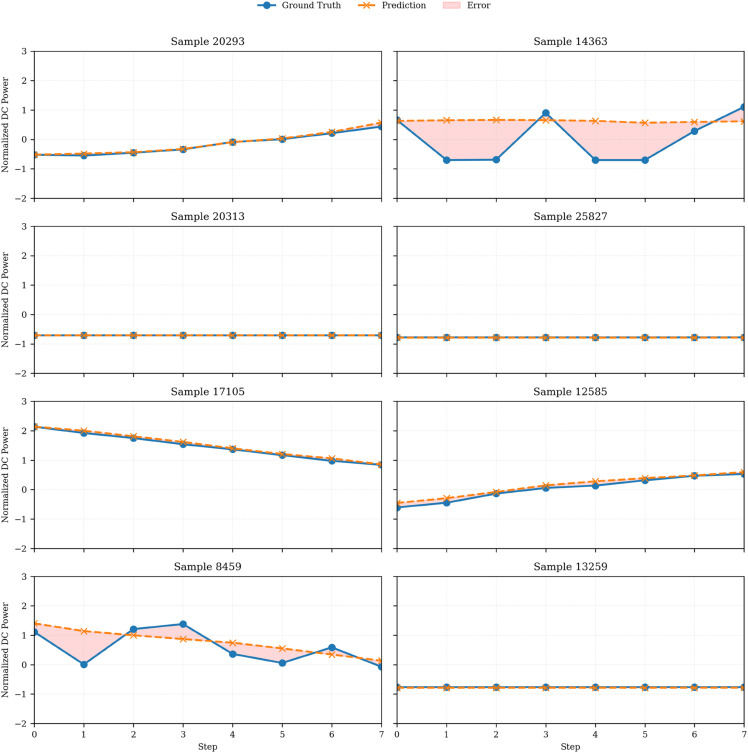
Prediction vs. ground truth DC power (8 steps ahead) on combined datasets.

**Fig 11:**
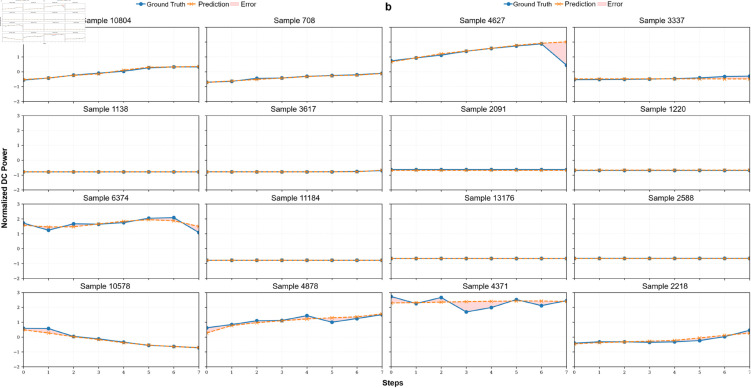
Test sample predictions and ground truth DC power across two solar plants.

For the forecast grid in Fig 10 and Fig 11, we randomly selected 8 test instances using a uniform sampling strategy across Plant 1 and Plant 2 data, aiming to illustrate diverse and representative behaviors across forecast horizons. Fig 10 depicts a comparison between predicted and actual DC power outputs for a representative inverter. The model captures short-term dynamics and power ramp-up trends effectively. Similar forecast plots are shown in Fig 11a and Fig 11b on Plant 1 and Plant 2 datasets, respectively.

Experiments were conducted to evaluate the performance of common baseline models such as LSTM, GRU, BiLSTM, and CNN-LSTM on the combined dataset. Basic architecture is used for these models. The optimizer, learning rate, decay rates, and number of epochs are kept the same for models during training. The dataset is processed in the same way as well. The proposed model performance comparison of the test set of the combined dataset is presented in Table 6. It is observed that SolarTrans demonstrates the best performance, but it comes at the cost of increased computational complexity. Compared to lightweight baselines like LSTM, GRU, and CNN-LSTM, which operate with relatively fewer parameters, SolarTrans employs stacked self-attention layers with high-dimensional feedforward blocks and multi-head configurations, resulting in a significantly larger model footprint. Despite this, the performance gains justify the added complexity for applications where forecasting precision is paramount. Future work may explore model compression techniques to improve deployment efficiency without compromising accuracy.

**Table 6:** Performance comparison of SolarTrans model on combined dataset.

Model	MAE	RMSE	R^2^
LSTM (2 layers, 32 hidden units)	0.1288	0.3430	0.8806
GRU (2 layers, 32 hidden units)	0.1390	0.3613	0.8674
BiLSTM (2 layers, 32 hidden units)	0.1454	0.3666	0.8638
CNN-LSTM (1 CNN layer with 32 filters, 1 LSTM layer with 32 hidden units)	0.1412	0.3562	0.8716
SolarTrans	0.1105	0.3189	0.8967

While the proposed model demonstrates strong performance on the current dataset, its generalization to other geographical sites and extended periods remains an important consideration. The use of per-inverter normalization helps reduce inter-device variability, and the inclusion of time-based features (hour, day, weekday, month) captures periodic and seasonal trends without reliance on plant-specific metadata. However, since the data originates from two co-located plants in a single region, it may not fully reflect the variability encountered across diverse climates, latitudes, or panel configurations. Differences in irradiation patterns, module technologies, and maintenance regimes can significantly affect power output dynamics. To improve generalizability, future work should focus on multi-site datasets and evaluate model robustness via cross-location validation. Additionally, transfer learning and domain adaptation strategies hold promise for adapting forecasting models to unseen environments with limited local data.

### Explainable forecasting via natural language generation

To enhance interpretability, we utilized a lightweight LLM module (Flan-T5) trained on synthetic explanations derived from rule-based logic. The module was capable of generating coherent natural language descriptions of solar forecasts based on irradiance, temperature, and temporal context. The rule-based explanation engine maps the physical input features such as irradiance, ambient temperature, module temperature, and forecasted DC power trends into human-readable narratives. The module identifies conditions like nighttime, high irradiance, or thermal inefficiency and contextualizes their impact on solar generation. To scale this into a natural language generation task, we collected structured input-output pairs and fine-tuned FLAN-T5-Base on Kaggle using dual NVIDIA T4 GPUs (T4×2). The dataset was split into train, validation, and test sets, and explanations were generated via a transformer model trained for 3 epochs. To evaluate the quality of the generated natural language explanations, we employed Recall-Oriented Understudy for Gisting Evaluation (ROUGE) [17] and Bilingual Evaluation Understudy (BLEU) [18], two widely used metrics in natural language generation tasks, particularly in summarization and translation. The explanation module is fine-tuned using the Hugging Face Transformers library using a batch size of 8 with a learning rate 1*e*–5 and weight decay of 0.001. Model checkpoints are saved at each epoch and evaluated using ROUGE and BLEU metrics.

The ROUGE metric evaluates the overlap between generated and reference explanations at the n-gram level. Specifically, ROUGE-N measures recall-based n-gram matches, while ROUGE-L captures the Longest Common Subsequence (LCS) alignment between the two texts. ROUGE-N is defined in Eq 3:

$$ROUGE\text{-}N=\frac{\sum_{S\in \{\text{Reference}\}}\sum_{{\text{gram}}_{N}\in S}\min({Count}_{\text{match}},{Count}_{\text{ref}})}{\sum_{S\in \{\text{Reference}\}}\sum_{{\text{gram}}_{N}\in S}{Count}_{\text{ref}}}.$$ (3)

where ${\text{Count}}_{\text{match}}$ is the number of overlapping n-grams between the candidate and reference, ${\text{Count}}_{\text{Ref}}$ is the total number of n-grams in the reference, and *S* refers to a reference sentence or summary within the set of reference texts. In practice, ROUGE-1 and ROUGE-L scores above 0.5 are generally considered strong for short, domain-specific explanations, indicating high content coverage and sequence similarity. Whereas, BLEU evaluates the precision of n-gram overlaps between the generated and reference text. It is defined as:

$$\text{BLEU}=\underset{\text{Brevity Penalty (BP)}}{\underbrace{\min\left(1,e^{1-\frac{r}{c}}\right)}}\cdot \exp\left(\sum_{n=1}^{N}w_{n}\log p_{n}\right)$$ (4)

where *r* is effective reference corpus length (sum of closest reference lengths per sentence)*c* is total candidate corpus length *w*_*n*_ indicates Uniform weights (typically $w_{n}=\frac{1}{N}$ for *N*-grams), *N* represents maximum *n*-gram order, and *p*_*n*_ is the modified n-gram precision. For short-form, factual explanations, BLEU scores in the range of 30–40 or higher typically indicate good alignment with the reference text. These metrics help quantify whether the model’s generated rationales are consistent, relevant, and faithful to domain-specific ground truth templates. To supplement these automatic metrics, we also provide qualitative examples comparing the generated explanations with human-crafted references.

Table 7 shows the performance of the fine-tuned FLAN-T5-based explanation model called Flan-T5-Solar when evaluated on 100 test samples using ROUGE and BLEU metrics. ROUGE measures the lexical overlap between generated and reference texts, emphasizing recall. We reported ROUGE-1 and ROUGE-2, which capture unigram and bigram overlaps, respectively, as well as ROUGE-L and ROUGE-Lsum, which consider the longest common subsequence to reflect sentence-level structural similarity. The high ROUGE-1 score of 0.7889 and ROUGE-L of 0.7759 indicate that the generated explanations align closely with the reference texts in both vocabulary and phrasing. In addition, BLEU evaluates the precision of n-gram matches between the generated text and the reference [18], penalizing overly short outputs through a brevity penalty.

**Table 7:** Evaluation metrics for FLAN-T5-Solar.

Metric	Score
ROUGE-1	0.7889
ROUGE-2	0.7211
ROUGE-L	0.7759
ROUGE-Lsum	0.7771
BLEU	0.6558

**Fig 12:**
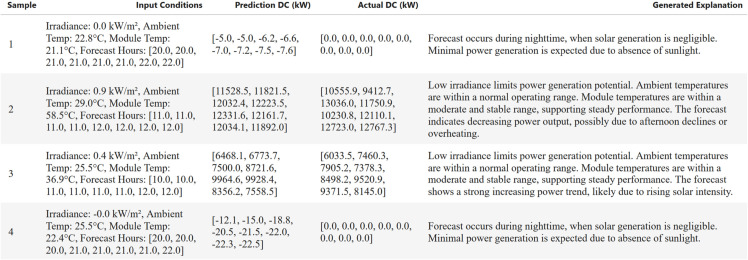
Samples of generated AI explanation for the predicted DC power.

Fig 12 presents a set of generated AI explanations corresponding to different environmental conditions and predicted DC power values. The examples demonstrate the model’s ability to generate coherent and context-sensitive narratives across a variety of scenarios. For instance, during nighttime hours when irradiance drops to near zero, the model correctly identifies the lack of sunlight and produces concise justifications indicating negligible solar output. In contrast, under low to moderate irradiance during daylight hours, the generated explanations adapt by acknowledging environmental conditions, such as ambient and module temperature, and relate them to potential fluctuations in output power. For Fig 12, we selected a fixed consecutive block of test samples, starting from the randomly selected sample in the test set.

**Fig 13:**
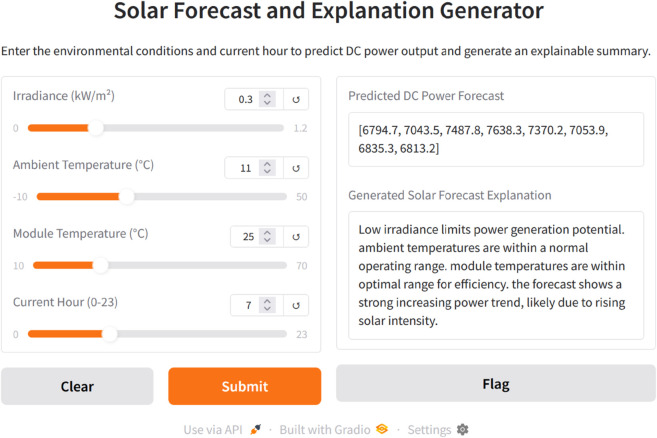
Screenshot of the interface for prediction and AI-generated explanation.

To demonstrate the real-time applicability of the SolarTrans model and its explainability component, we developed an interactive web interface using Gradio [19], hosted within a Kaggle Notebook environment. Gradio is a widely used open-source Python library for building machine learning interfaces. This interface enables users to input environmental variables, including irradiance, ambient temperature, module temperature, and forecast hour, and receive both a multi-step DC power prediction and a corresponding AI-generated natural language explanation. The interface serves as a proof of concept, illustrating how explainable solar forecasting can be operationalized for end-users, such as researchers, engineers, or energy planners. Fig 13 also shows a screenshot of the web interface for interactive use. The user inputs irradiance, ambient temperature, module temperature, and the current hour, after which the model outputs a multi-step DC power forecast along with an automatically generated natural language explanation.

### SHAP visualization using SolarTran model

**Fig 14:**
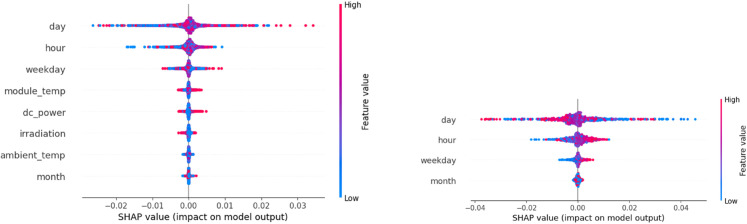
SHAP summary plots.

We applied SHAP analysis to interpret the predictions of our SolarTrans model. Fig 14 shows SHAP summary plots for encoder (left) and decoder (right) inputs. Each point represents a SHAP value for a feature at a specific timestep across the test set. Color indicates feature value (red = high, blue = low). Features are ranked by average impact on model output. The SHAP summary plot reveals that among encoder inputs, temporal features such as day and hour have the highest influence on model predictions, followed closely by weekday and module-level variables, including Module temp, DC Power, and Irradiation. This ranking suggests that short-term temporal patterns, especially day-of-month and hour-of-day, are more predictive of solar power generation than weather variables alone.

**Fig 15:**
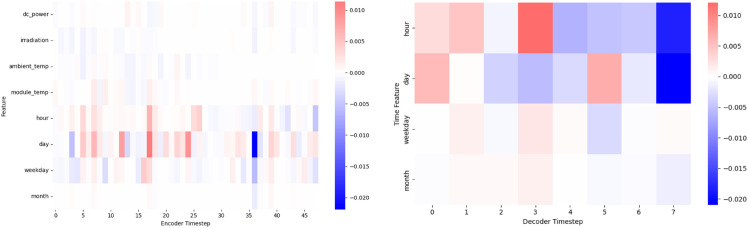
Temporal distribution of features using SHAP heatmaps.

We also visualized the temporal distribution of feature attributions using SHAP heatmaps (Fig 15). The encoder heatmap highlights concentrated influence from time-related features (hour, day) and occasional bursts from irradiation and DC Power, suggesting event-driven attention. The decoder heatmap shows structured importance patterns across forecast steps, particularly in early hours, supporting the model’s ability to condition predictions on time-aware trends. These patterns confirm that SolarTrans effectively leverages both temporal and weather signals across input sequences.

## Conclusion

This paper presents a hybrid deep learning framework that combines a Transformer-based architecture (SolarTrans) for short-term solar power forecasting with a fine-tuned large-language model (Flan-T5-Solar) for generating natural language explanations. By leveraging multivariate environmental and operational inputs along with temporal covariates, SolarTrans delivers accurate multi-horizon predictions of DC power output across heterogeneous inverter sites. The model architecture incorporates learnable positional encodings, causal masking, and per-inverter normalization to improve generalization and temporal fidelity. To enhance interpretability, a complementary Flan-T5-Solar module was integrated to generate context-aware, human-readable explanations of the forecasts. This dual-stage pipeline supports both numerical accuracy and transparent post-hoc reasoning, enabling more actionable insights for grid operators and solar asset managers.

Experimental results demonstrate that the proposed approach achieves low forecasting error while offering meaningful explanatory narratives grounded in physical features such as irradiance and temperature. The modular design further enables easy extensibility to new sites or sensors. Future work includes exploring probabilistic extensions to quantify uncertainty in forecasts, integrating additional atmospheric features (e.g., cloud cover, humidity), and deploying the framework in real-time operational settings to evaluate its effectiveness under dynamic grid conditions.
